# Oxidative Stress in Patients with Drug Resistant Partial Complex Seizure

**DOI:** 10.3390/bs8060059

**Published:** 2018-06-09

**Authors:** Lourdes Lorigados Pedre, Juan M. Gallardo, Lilia M. Morales Chacón, Angélica Vega García, Monserrat Flores-Mendoza, Teresa Neri-Gómez, Bárbara Estupiñán Díaz, Rachel M. Cruz-Xenes, Nancy Pavón Fuentes, Sandra Orozco-Suárez

**Affiliations:** 1 Immunochemical Department, International Center for Neurological Restoration, 25th Ave, Playa, Havana 15805, Cuba; lourdesl@neuro.ciren.cu (L.L.P.); nancy@neuro.ciren.cu (N.P.F.); 2 Medical Research Unit in Nephrological Diseases, Specialty Hospital, National Medical Center “XXI Century”, IMSS, Mexico City 06720, Mexico; jmgallardom@gmail.com; 3 Clinical Neurophysiology Lab., International Center for Neurological Restoration, 11300 Havana, Cuba; lily@neuro.ciren.cu; 4 Nanomaterials Laboratory, Research Center in Health Sciences, Autonomous University of San Luis Potosí, San Luis Potosi 78300; Mexico; tnerigomez@gmail.com; 5 Morphological Laboratory, International Center for Neurological Restoration, Havana 11300, Cuba; baby@neuro.ciren.cu; 6 Biology Faculty, University of Havana, Havana 10400, Cuba; rachel.cruz@fbio.uh.cu; 7 Medical Research Unit in Neurological Diseases, Specialty Hospital, National Medical Center, XXI Century, IMSS, Mexico City 06720, Mexico; ange_li_k@hotmail.com (A.V.G.); moonsefm@hotmail.com (M.F.-M.)

**Keywords:** oxidative stress, drug-resistant epilepsy, redox, MDA, 4-HNE, 3-NT, AGEs, SOD, nitric oxide, vitamin C

## Abstract

Oxidative stress (OS) has been implicated as a pathophysiological mechanism of drug-resistant epilepsy, but little is known about the relationship between OS markers and clinical parameters, such as the number of drugs, age onset of seizure and frequency of seizures per month. The current study’s aim was to evaluate several oxidative stress markers and antioxidants in 18 drug-resistant partial complex seizure (DRPCS) patients compared to a control group (age and sex matched), and the results were related to clinical variables. We examined malondialdehyde (MDA), advanced oxidation protein products (AOPP), advanced glycation end products (AGEs), nitric oxide (NO), uric acid, superoxide dismutase (SOD), glutathione, vitamin C, 4-hydroxy-2-nonenal (4-HNE) and nitrotyrosine (3-NT). All markers except 4-HNE and 3-NT were studied by spectrophotometry. The expressions of 4-HNE and 3-NT were evaluated by Western blot analysis. MDA levels in patients were significantly increased (*p* ≤ 0.0001) while AOPP levels were similar to the control group. AGEs, NO and uric acid concentrations were significantly decreased (*p* ≤ 0.004, *p* ≤ 0.005, *p* ≤ 0.0001, respectively). Expressions of 3-NT and 4-HNE were increased (*p* ≤ 0.005) similarly to SOD activity (*p* = 0.0001), whereas vitamin C was considerably diminished (*p* = 0.0001). Glutathione levels were similar to the control group. There was a positive correlation between NO and MDA with the number of drugs. The expression of 3-NT was positively related with the frequency of seizures per month. There was a negative relationship between MDA and age at onset of seizures, as well as vitamin C with seizure frequency/month. We detected an imbalance in the redox state in patients with DRCPS, supporting oxidative stress as a relevant mechanism in this pathology. Thus, it is apparent that some oxidant and antioxidant parameters are closely linked with clinical variables.

## 1. Introduction

Oxidative stress (OS) is a biochemical state in which reactive oxygen species (ROS) are generated. Since the 1970s, OS been associated with diverse physiological and pathological conditions, including epilepsy. At high concentrations, ROS react readily with proteins, lipids, carbohydrates, and nucleic acids, often inducing irreversible functional alterations or even complete destruction. OS is defined as an imbalance between oxidants, nitrosative stress and antioxidants, which results in a relative or actual excess of oxidative species, and this leads to disruptions in signaling, redox control, and/or molecular damage. OS is also involved in acute and chronic central nervous system (CNS )injury and is a major factor in the pathogenesis of neuronal damage [1]. OS is also involved in acute and chronic CNS injury and is a major factor in the pathogenesis of neuronal damage [2]. 

Epilepsy is one of the most common and severe brain disorders in the world, affecting at least 50 million people worldwide. It is characterized by recurrent spontaneous seizures due to an imbalance between cerebral excitability and inhibition, with a tendency towards uncontrolled excitability. Approximately, 60–80% of patients with epilepsy can be controlled with antiepileptic drugs [3]. In more than 60% of all cases, seizures remit permanently. Nevertheless, a substantial proportion of patients (30%) do not respond to antiepileptic drug medication, despite administration in an optimally monitored regimen. Such cases are often loosely termed drug-resistant, intractable or pharmacoresistant [4]. The majority of these patients suffer from the focal form of epilepsy. The areas of epileptogenesis in these cases are usually characterized by cell loss [5,6].

Emerging works have indicated the involvement of redox imbalance in epileptogenesis [7]. Increased oxidant generation has been demonstrated to be induced in epilepsy by recurrent seizures with high levels of OS biomarkers and low antioxidant defenses present in epileptic subjects [8]. However, whether OS is a consequence, a causative factor, or both, in mechanisms involved in seizures is not clear [9,10].

Accumulating evidence supports the association between OS and seizures in seizure generation and in the mechanisms associated with refractoriness to drug therapy. Alterations in the antioxidant enzymes [11,12,13,14] and increases in the indicators of oxidative damage to biomolecules, such as malondialdehyde (MDA), protein carbonyls and 8-hydroxy-2-deoxyguanosine and activation of nicotinamide adenine dinucleotide phosphate oxidase have been reported [10,12,13]. Similarly, data from animal studies suggests that prolonged seizure activity might result in increased production of ROS. Further, the generation of nitric oxide and peroxynitrite has been shown to precede neuronal cell death in vulnerable brain regions [15,16].

Despite the alterations in the redox state described in epilepsy, little is known about how they behave jointly in complex partial seizures that are refractory to drugs in terms of parameters, such as the expression of 3-nitrotirosine (3-NT), 4 hydroxynoneal (4-HNE), levels of oxidants and antioxidants, and their relationships with clinical parameters, such as duration of epilepsy, frequency of seizures and number of drugs (poly or monotherapy).

Neuronal cells in the brain are highly sensitive to oxidative stress; therefore, the prolonged excitation of neurons during seizures can lead to injury resulting from biochemical alterations and specifically, to the role played by the oxidation state [17]. Excessive ROS generation can cause damage to neuronal cells, inducing cell death via either apoptotic or necrotic pathways [18]. Taking into account this information and based on the results described previously by our group, in which apoptotic and necrotic death were evidenced in temporal lobe epileptic patients [19,20], we proposed the evaluation of oxidative stress in patients who were suffering from drug-resistant partial complex seizure (DRPCS).

There have been few studies that have evaluated markers of oxidative stress in DRPCS in terms of parameters such as the expression of 3-nitrotirosine (3-NT), 4 hydroxynoneal (4-HNE), markers of damage to proteins, lipids and advanced glycation products (AGEs), and nitric oxide (NO) as well as antioxidants (superoxide dismutase (SOD), gluthatione, vitamin C and uric acid). The main objective of this work is not only to evaluate a large number of oxidative stress markers but also to explore the possible relationship of these markers with clinical parameters, such as the time of crisis evolution, the number of seizures per month and the medication received by these patients, e.g., monodrug or multidrug therapy.

## 2. Materials and Methods

### 2.1. Patient Information

The serum concentration of oxidative stress markers was studied in eighteen patients from the International Center for Neurological Restoration (CIREN) with DRCPS (6 females and 12 males, with mean age 33.28 ± 12.36 years). The criteria to be considered drug-resistant were the following: the presence of seizures for more than two years, two complex partial monthly seizures, two cycles of monotherapy and at least one cycle of polytherapy. All patients were evaluated in the Video-EEG Telemetry Unit from CIREN, and they underwent a complete general and neurological physical examination and anatomical evaluation by Magnetic Resonance Imaging (1.5 T MAGNETOM SINPHONY) and SPECT.

In terms of antiepileptic treatment, the most commonly used antiepileptic drugs (AEDs) by patients in the study were carbamazepine (3–6 tablets daily), followed by lamotrigine (2–3 tablets daily) and magnesium valproate (1–6 tablets daily).

To correlate OS markers with the number of medications taken by patients, treatments were grouped as follows: monodrug therapy (a single AED) or multidrug therapy (two or three AEDs).

The collected clinical parameters included demographic characteristics and clinical states, as shown in Table 1. The parameters were age, gender, time of seizure evolution, frequency of seizure by month and seizure localization and medication.

### 2.2. Samples

Venous blood samples (5 mL) were taken from the patients by antecubital puncture after asepsis of the region. The blood was centrifuged, and the serum was kept at −20 °C until its use. The serum samples were used to analyze the following routine biochemical parameters and oxidative biomarkers: lipid peroxidation measured by MDA, advanced oxidation protein products (AOPP), advanced glycation end products (AGEs); nitric oxide (NO), 3-NT, 4-HNE, uric acid, vitamin C and SOD.

Patients with chronic medical diseases (hypertension, diabetes, collagen vascular diseases, rheumatoid arthritis, other neurological diseases), those who were smokers, alcoholics, and those taking any other medications were excluded from the study.

The evaluation included analyses of seizure semiology, video-EEG monitoring using noninvasive methods, anatomical neuroimaging (MRI) and functional neuroimaging (SPECT, functional MRI) and neuropsychological assessment. None of the patients or controls received any antioxidant supplementation.

The control group was formed by 80 healthy volunteers who were aged 31.3 ± 8.64 including 50 males and 30 females matched to patients group. It was taken into account that the supposedly healthy subjects did not show antecedents of neurological diseases, were not smokers, alcoholics and did not take any medication or medical supplements.

### 2.3. Determination of Lipid Peroxidation

Lipid peroxidation was assessed by measuring MDA using the thiobarbituric acid (TBA)-reactive substances test [21] and was calculated in µmol/L. To an aliquot of plasma, 200 µL of 25% trichloroacetic acid was added. The samples were incubated at 4 °C for 15 min, followed by centrifugation at 4 °C, 5000× *g* for 3 min, and the supernatant (100 µL) was neutralized with 4 M NaOH (JT Baker, Xalostoc, Edo. De Mexico, Mexico). Then, to 1 mL of the above solution, 1 mL of 0.7% TBA (Acros Organics, Belgium) was added. This mixture was incubated at 90 °C for 60 min. The color reaction was measured spectrophotometrically (532 nm) in the organic phase (1-butanol, Sigma-Aldrich, St. Louis, MO, USA). Tetramethoxypropane was used as a standard. The results were expressed as TBARs per µmol/L.

### 2.4. AOPP Assay

AOPP were measured by spectrophotometry on a microplate reader (Multiskan, Thermolab) and were calibrated with chloramine-T solutions (Sigma, St. Louis, MO, USA) which, in the presence of potassium iodide, is absorbed at 340 nm [22]. Two hundred microliters of plasma diluted in a ratio of 1:5 in PBS was placed on a 96-well microtiter plate, and 20 μL of acetic acid was added. In standard wells, 10 µL of 1.16 M potassium iodide (Sigma, St. Louis, MO, USA) was added to 200 μL of chloramine-T solution (0–100 μmol/L) followed by 20 μL of acetic acid. The absorbance of the reaction mixture was immediately read at 340 nm on the microplate reader against a blank containing 200 μL of PBS, 10 μL of potassium iodide, and 20 μL of acetic acid. The chloramine-T absorbance at 340 nm was linear within the range of 0 to 100 μmol/L. AOPP concentrations were expressed as micromoles per liter of chloramine-T equivalent. AOPP were expressed in umol/L.

### 2.5. Advanced Glycation End Products AGEs

AGE concentrations were measured after the blood had been centrifuged. For fluorescent AGEs, we employed a 96-plate spectrophotofluorimeter (Fluoroskan Ascent FL. Vantaa, Finland). Briefly, 100 μL of serum was deproteinized with TCA (ReactivosQuimica Meyer, Mexico City, Mexico) at a concentration of 300 mmol/L. Then, 200 μL chloroform was added, vortexed for 60 s and centrifuged at 14,000 rpm. Finally, 200 μL of the respective supernatant was placed in each well, in triplicate fluorescence. The intensity was read at 440 nm after excitation at 355 nm. Results were expressed as arbitrary units (AU) corrected by serum proteins (measured by absorptiometry at 280 nm). The intra-assay variation coefficient was 8.4%. To ensure adequate readings by the spectrophotofluorimeter, fluorescence calibration curves were performed using quinine sulfate as the standard, which has similar excitation and emission spectra (360 and 440 nm, respectively).

### 2.6. Nitric Oxide

NO was measured by determining the total quantity of nitrite (NO_2_^−^), which is the stable product of NO metabolism in plasma. Griess reagent was used (an aqueous solution of 1% sulfanilamide (Sigma-Aldrich, St. Louis, MO, USA) with 0.1% naphthylethylenediamine (Sigma-Aldrich, St. Louis, MO, USA) in 2.5% H_3_PO_4_ (2.5%, JT Baker, Xalostoc, Mexico), which forms a stable chromophore with NO_2_^−^ and absorbs light at 546 nm (Green LC 1982). The calibration curve was constructed using different concentrations of sodium nitrite dissolved in 0.9% NaCl. The NO level was expressed in µmol/L.

### 2.7. Western Blot Analysis for 3-Nitrotyrosine and 4-Hidroxynonenal

The samples of serum were quantified according to the Bradford method (Bio-Rad, Hercules, CA, USA). Proteins (12 μg) were separated by electrophoresis on 10% SDS–PAGE gel at 80 V. Gels were transferred to Immun-Blot PVDF membrane for protein blotting (Bio-Rad) at 20 V at room temperature for 1 h. Membranes were blocked with blocking buffer (Millipore, Burlington, MA, USA) and incubated at 4 °C overnight with primary antibodies diluted in a ratio of 1:3000. Pre-stained broad range markers (BioRad, CA, USA) were included for size determination.

The following primary antibodies were used: goat anti-4-hidroxynonenal polyclonal antibody (AB5605 Millipore, Burlington, MA, USA), rabbit anti-3-NT polyclonal antibody, and rabbit anti-transferrin polyclonal antibody (Santa Cruz Biotechnology, CA, USA). After incubation with the primary antibody, membranes were washed and incubated with horseradish peroxidase-coupled secondary antibodies (VECTOR, diluted 1:15,000). Immunoreactive bands were detected by using an enhanced chemiluminescence system (Clarity Western ECL substrate by BIO-RAD). Membranes were stripped by employing a commercial solution (Millipore, Burlington, MA, USA) retested with anti-transferrin polyclonal antibody and detected by the system mentioned above. Anti-transferrin was used to correct the differences in the total amount of loaded protein. The intensity of the protein bands was quantified by densitometry using a molecular imager Fusion FX VilberLourmat (Vilber, Marne-la-Vallée, France), and captured data was quantified and analyzed by densitometry using Quantity One Image Analysis Software. The density of each band was normalized to its respective loading control (transferrin) and was expressed as the integrated density value.

### 2.8. SOD Activity

SOD was determined by the Marklund and Marklund method [23]. The anionic superoxide radical participates in the autoxidation of pyrogallol. To this end, a pyrogallol solution was prepared in HC1 (JT Baker, Xalostoc, Mexico) and incubated at 40 °C. To 50 μL of sample, 200 μL of a mixture of Tris-EDTA-HC1 was added and read at 420 nm in a DU 50 spectrophotometer (Beckman, Palo Alto, CA, USA). Subsequently, the pyrogallol solution was added and the increase in absorbance was re-measured every 30 s for 3 min. The reagent blank was made in the same way but distilled water was used instead of sample. The activity of SOD is expressed in U/gHb.

### 2.9. Vitamin C

The measurement of vitamin C was done according Prieto et al. [24]. This method is based on the reduction of molybdate (VI) to molybdate (V) by the sample, followed by the formation of a complex between phosphate and molybdate (V), which has an optimal absorption at 695 nm. The calibration curve was prepared with ascorbic acid and read at 695 nm. The vitamin C concentration is expressed in mmol/L.

### 2.10. Uric Acid

Uric acid is a metabolite of purines, nucleic acids and nucleoproteins. Usually, the concentration of uric acid in serum varies from one individual to another according to various factors, such as sex, diet, ethnicity, genetic makeup, pregnancy, and many other conditions. Uric acid was measured using the enzymatic Uricostat reagent kit (Wiener brand, Rosario, Argentina), following the manufacturer’s instructions. The levels of uric acid are expressed in mg/dL.

### 2.11. Gluthatione

The activity of glutathione was analyzed using the method of Beutler, et al. [25]. Gluthatione was measured with 5,5 ‘dithiobis- (2-nitrobenzoic acid) (DTNB). The reaction mixture contained 1 mL of gluthatione (Amresco), 2 mmol of buffer, 400 mmol of PBS (pH 7.0), 4 mmol of EDTA, 1 mmol of 0.5% sodium azide, 250 μL of seminal fluid or sperm and bidistilled water to give a total of 4 mL. After incubation at 37 °C for five minutes, 1 mL of prewarmed T-BOOH at a concentration of 1.25 mmol was added and re-incubated for four more minutes. At the end of that period, 1 mL was recovered, and 4 mL of phosphoric acid was added, centrifuged at 2000× *g*, at room temperature, for 10 min. Two milliliters of the supernatant was recovered, and 2 mL of Na_2_HPO_4_ 400 mmol and 1 mL of the DTNB reagent were added. The absorbance was measured at 412 nm. The targets and standards were prepared similarly. The activity of glutathione was expressed as U/mg of protein.

### 2.12. Ethical Considerations

All procedures followed the rules of the Declaration of Helsinki of 1975 for human research, and the study was approved by the Ethics Committee of the International Center for Neurological Restoration (Record 03/2015).

### 2.13. Statistical Processing

Statistical analysis was carried out using GraphPad Prism 5 software (GraphPad Software, Inc., La Jolla, CA, USA). The values are expressed as means ± SEMs. Normal distribution and homogeneity of variance of the data were tested with the Kolmogorov–Smirnov and Levene tests, respectively. The comparisons between two groups were made by means of the t-Student test. The Spearman correlation was used for the correlation study. In all cases, statistically significant differences were considered when *p* ≤ 0.05.

## 3. Results

There has been previous evidence supporting a gender difference in epilepsy related to OS markers [26,27]. In this study there were no gender differences for all OS markers evaluated, so the concentrations in males and females were unified into one group for each marker.

### 3.1. Proteins and Lipid Damage and Advanced Glycation

**Figure 1:**
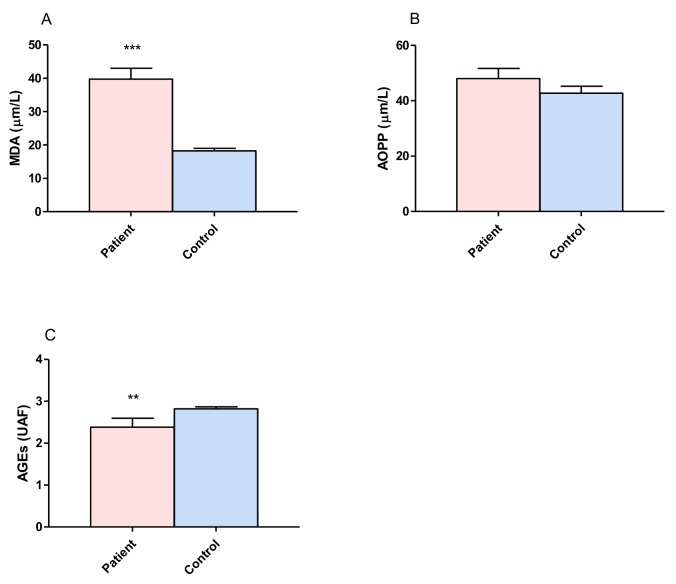
Comparison of concentrations of malondialdehyde (MDA), advanced oxidation protein products (AOPP) and advanced glycation end products (AGEs) between patients with drug resistant partial complex seizure and controls. (**A**) MDA serum concentration. (**B**) AOPP serum concentration. (**C**) AGEs serum concentration. The bars represent the means ± the standard errors of the mean. Unpaired t-Student test. ** *p* ≤ 0.004, *** *p* ≤ 0.0001.

The brain has a high lipid content, and its oxygen consumption and oxidative metabolism make it susceptible to oxidative stress. Lipid peroxidation involves the oxidative degradation of lipids. Protein oxidation is irreversible oxidative damage, and protein damage is considered to be a marker for severe oxidative stress. In order to evaluate the damage to proteins and lipids, we measured the concentrations of MDA, AOPP and AGEs in DRPCS patients and compared these with the control group (Figure 1). The results showed a statistically significant increase in MDA (*p* ≤ 0.00001, Figure 1A) in patients (39.78 ± 3.23 µm/L) when compared with controls (18.23 ± 0.81 µm/L). No differences were found between groups with regard to AOPP; however, it is possible to observe a trend towards the steric increment in these products (patients: 48.04 ± 3.61 µm/L, controls: 42.73 ± 2.53 µm/L Figure 1B). There was a significant decrease in AGEs (*p* ≤ 0.0049, Figure 1C) in patients (2.38 ± 0.21 UAF) versus controls (2.82 ± 0.04 UAF).

### 3.2. Nitric Oxide

**Figure 2:**
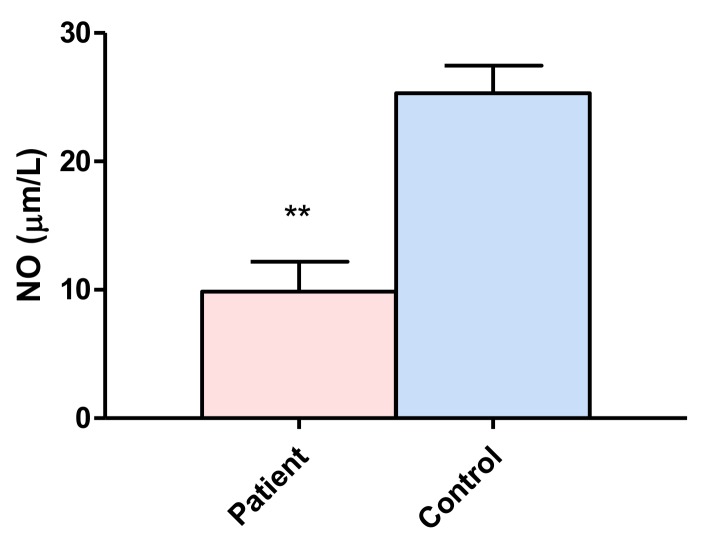
Comparison of nitric oxide (NO) concentrations between patients with drug resistant partial complex seizure and controls. The bars represent the means ± standard errors of the mean. Unpaired t-Student test. ** *p* ≤ 0.0054.

Nitric oxide is a free radical that is formed biologically through the oxidation of L-arginine by nitric oxide synthase. The NO levels in patients (9.85 ± 2.34 µm/L) were lower than those of the control group (25.31 ± 2.14 µm/L), with a significant level of *p* ≤ 0.0054 (Figure 2).

### 3.3. Expression of 4-Hydroxy-2-Noneal and 3-Nitrotyrosine

**Figure 3:**
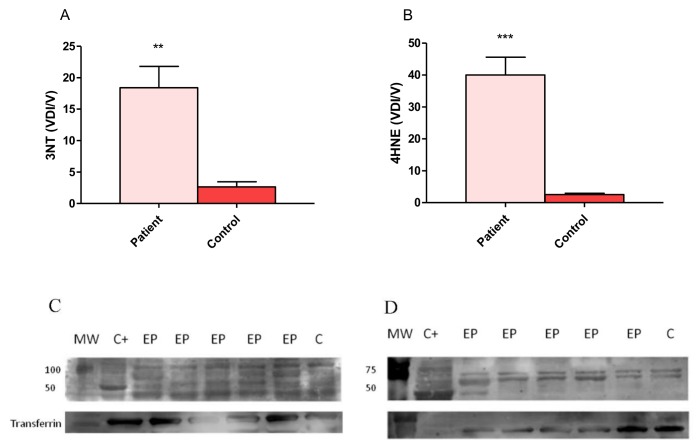
3-Nitrotyrosine (3-NT) and 4-hydroxy-2-nonenal (4-HNE) expression in patients with drug resistant partial complex seizure. (**A**) Representative example of the blot of 3-NT in a patient and transferrin as a control protein. (**B**) Representative example of the blot of 4-HNE in a patient and transferrin and controls. (**C**) Comparison of relative optical density values of 3-NT in patients and control subjects. (**D**) Comparison of relative optical density values of 4-HNE in patients and control subjects. The bars represent the means ± the standard errors of the mean, unpaired t-Student test, ** *p* ≤ 0.003, *** *p* ≤ 0.0001. C+ rat protein extract was used as the positive control in both blots. MW; molecular weight, EP; epileptic patient, C; control.

3-NT is an oxidative marker of NO inactivation, which, in our results, showed a statistically significant increase in the expression of 3-NT in group of patients with DRCPS (18.40 ± 3.39) compared with the control subjects (2.65 ± 0.79, Figure 3A).

The expression of 4-HNE (one of the major end products of lipid peroxidation) in patients (40.04 ± 5.53) was increased (*p* ≤ 0.00001) compared with the control group (2.52 ± 0.39, Figure 3B).

### 3.4. Antioxidant Evaluation

**Figure 4:**
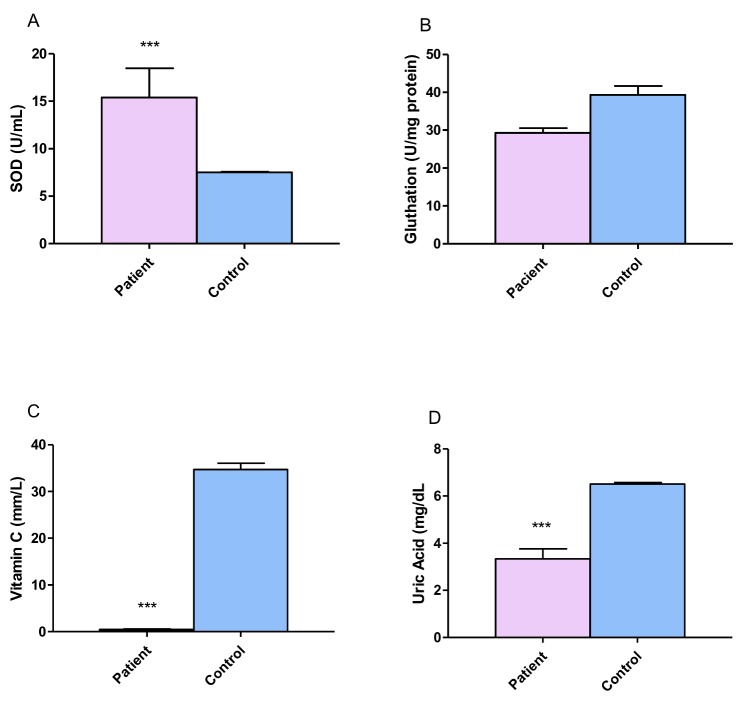
Comparison of superoxide dismutase (SOD), glutathione, vitamin C and uric acid concentrations between patients with drug resistant partial complex seizure and controls. (**A**) SOD serum concentration. (**B**) Gluthatione serum concentration. (**C**) Vitamin C serum concentration and (**D**) Uric acid serum concentration. The bars represent the means ± standard errors of the mean. Unpaired t-Student test. *** *p* ≤ 0.0001.

In order to evaluate the serum concentrations of antioxidants in DRPCS patients, we measured the concentrations of SOD, glutathione, vitamin C and uric acid. The results showed a statistically significant increase in SOD (*p* ≤ 0.00001, Figure 4A). There were no differences between the glutathione levels in patients versus the control group (*p* ≤ 0.07); however, a tendency to decrease this parameter in patients was observed (Figure 4B). Significantly lower values of vitamin C and uric acid were shown in patients (3.33 ± 0.42 mm/L, 6.51 ± 0.05 mg/dL, Figure 4C,D respectively.) when compared with control group (34.72 ± 1.32 mm/L, 6.51 ± 0.05 mg/dL respectively).

### 3.5. Correlation of Oxidative Stress Parameters with Clinical Data

Table 2 shows the correlation between the parameters of oxidative stress evaluated in this study with clinical data such as the time evolution of seizures, the frequency of seizures per month and the number of drugs taken by a patient. There was a positive correlation between MDA and NO with the number of drugs. Patients with multidrug treatments had the highest levels of MDA and NO, while the elevation of MDA was related to the early age of onset of seizures. Similarly, the frequency of crises per month were negatively related to vitamin C and positively related to the expression of 3-NT.

## 4. Discussion

The molecular mechanisms that lead to seizures and epilepsy are not well understood. Previous studies have demonstrated that seizure-induced mitochondrial dysfunction and excess free radical production cause oxidative damage to cellular components and initiate the mitochondrial apoptotic pathway [28,29]. OS is also considered an important consequence of excitotoxicity and inflammation, two of the proposed mechanisms for seizure-induced brain damage [20,29,30,31,32,33,34].

Previous studies have found increased activities of SOD, CAT, markers of lipid peroxidation and decreased activities of glutathione peroxidase (GPx) in pharmacoresistant temporal lobe epilepsy (TLE) patients [35,36]. Sudha K, (2001) reported a decrease in glutathione reductase. The lipid peroxidation and percentage hemolysis were higher compared to controls. Furthermore, erythrocyte glutathione reductase and plasma ascorbate and vitamin A concentrations were lower [8]. Meanwhile, many different studies have noted increased markers in lipid peroxidation [12,36]. On the other hand, there have also been other studies that have not detected changes in SOD, CAT, GPx and glutathione reductase activities [8,36,37,38].

Our group has been studying the impact of epilepsy surgery on serum markers of oxidative damage in pharmacoresistant temporal lobe epilepsy (TLE) patients [12]. Before surgery, we found increased activities of SOD, CAT, markers of lipid peroxidation and decreased GPx activity. An interesting finding was the positive correlation between the duration of the disease and advanced oxidation protein product levels. This result suggests the early presence of oxidative damage to proteins in the initial stages of the illness. This could be due to protein repairing mechanisms that do not act as efficiently as in other biomolecules. After surgery, the patients showed a tendency for the studied variables to normalize, except for SOD activity. The outlying redox state of the patients markedly improved after surgery, which was clearly evidenced by decreases in MDA and advanced oxidative protein products levels two years after surgery. The recovery in GPx activity was also notorious, as it contributed to a decrease in oxidative damage and a better redox balance [12]. On the other hand, we can speculate that the sustained increase in superoxide dismutase activity could recede if the epileptic activity in the remaining regions eventually disappears in these patients. Finally, the increase in CAT activity levels seems to be a cellular response to the intense ROS production triggered by seizure episodes.

On the other hand, there is information that supports the suggestion that inflammation and oxidative stress are linked with a number of chronic diseases, including diabetes and diabetic complications, hypertension, cardiovascular diseases, neurodegenerative diseases, and aging [39,40,41,42]. Inflammatory cells liberate a number of reactive species at the site of inflammation, leading to exaggerated oxidative stress [43,44]. Secondly, a number of reactive oxygen/ nitrogen species can initiate intracellular signaling cascade that enhances proinflammatory gene expression [45]. There is a group of previous findings from our working group that supports the participation of inflammatory processes in drug-resistant epilepsy [46], which could be a source of the OS detected in these patients. Thus, inflammation and oxidative stress are closely related pathophysiological events that are tightly linked with one another.

### 4.1. Proteins, Lipid Damage and Advanced Glycation Products

Lipid peroxidation is one of the major sources of free radical-mediated injury that directly damages membranes and generates a number of secondary products. In particular, markers of lipid peroxidation have been found to be elevated in brain tissues and body fluids in several neurodegenerative diseases, such as epilepsy. This complex process involves the interaction of oxygen-derived free radicals with polyunsaturated fatty acids (PUFAs), resulting in a variety of highly reactive electrophilic aldehydes, including MDA, 4-HNE, and acrolein. Therefore, the evaluation of MDA levels in biological materials can be used as an important indicator of lipid peroxidation for various diseases. In the present study, we found significantly higher levels of MDA in patients with epilepsy compared to controls; these results are in agreement with previous studies in which it has been reported that a higher level of MDA is associated with epilepsy [47,48]. On the other hand, these studies showed that the levels of MDA were significantly increased in treated patients (with AED) compared to untreated patients, which was also observed in patients with epilepsy. There was a significant correlation with the number of drugs administered (Table 2) which suggests that additional oxidative stress was induced by AEDs, causing the recurrence of seizures and intolerance to drugs. Peroxidation of membrane lipids can have numerous effects, such as increased membrane rigidity, decreased activity of membrane-bound enzymes (e.g., sodium pump), altered activity of membrane receptors, and altered permeability [49]. In addition to effects on phospholipids, radicals can also directly attack membrane proteins and induce lipid–lipid, lipid–protein, and protein–protein cross-linking, all of which obviously have effects on membrane function [50]. Of these products, MDA, 4-HNE, and acrolein can cause irreversible modification of phospholipids, proteins, and DNA, resulting in impaired function and consequently, cell death, a fact observed in complex partial epilepsy, which has been demonstrated in previous works involving pharmacoresistant epilepsy patients [19,46]. On the other hand, the formation of AGEs, a group of modified proteins and/or lipids with damaging potential, is one contributing factor. However, it has been reported that AGEs increase reactive oxygen species formation and impair antioxidant systems, while the formation of some AGEs is induced under oxidative conditions. However, in the patients with epilepsy, the values were lower than those in controls; thus, AGEs contribute less to chronic stress conditions in epilepsy.

Lipid peroxidation alters membrane structure, affecting its fluidity and permeability and the activity of membrane-bound proteins and produces many cytotoxic and reactive by-products. Among these, 4-HNE is able to form adducts with biomolecules, including proteins, lipids and nucleic acids, thereby propagating oxidative damage [51]. HNE-mediated damage to proteins is a known oxidative posttranslational modification that leads to functional changes or deactivation of enzymes, transporters, ion channels and receptors [52].

In this study, the levels of damage to biomolecules increased and coincidentally, the expression of 4-HNE is high in the patients in relation to the control group. Furthermore, an increase in 4-HNE occurs in various pathological conditions, including neurological diseases, where it contributes to cell death and neurodegeneration [52,53]].

4-HNE is considered to be a “second toxic messenger” that can propagate and amplify initial oxidative injury. 4-HNE can form covalent bonds with three different amino acyl side chains, i.e., lysyl, histidyl, and cysteinyl residues. In addition, 4-HNE can modify protein structure through Schiff base formation with lysyl residues, leading to the formation of pyrrole, and/or form intra- and/or intermolecular cross-links. Due to its amphiphilic nature, hydroxyaldehyde can diffuse across membranes and covalently modify proteins in the cytoplasm and nucleus, far from their site of origin [52].

Our results support an increase in the expression of 4-NHE in patients with DRPCS, which coincides with that reported by other authors, such as Pecorelli et al., that describe high levels of 4-HNE-protein adduct brain tissues and body fluids in several neurological and neurodegenerative diseases and in drug-resistant epileptic patients [10,54,55]. This confirms the evidence of lipid peroxidation and indicates the presence of oxidative damage to proteins in human epileptic brain. Other studies have evaluated 4-HNE levels in epileptic diseases and reported high levels of brain 4-HNE during seizures in the kindling model [56]. In addition, it is well known that HNE can be an apoptotic inducer [57]. High levels of 4-HNE may eventually promote cell death; there is evidence that 4-HNE plays a pivotal role in neuronal death, as has already been demonstrated in several neurological diseases [53,58,59].

NO is a free radical that is formed biologically through the oxidation of L-arginine by nitric oxide synthase. The exact role of NO in the pathophysiology of epilepsy is still unclear. Several studies have shown that NO may act as an endogenous anticonvulsant [60,61,62], although some studies have shown that NO acts as a proconvulsant [63,64]. Our results showed a significant decrease in NO in patients compared with the control group and a relationship with the number of AEDs that patients receive.

On the other hand, 3-NT is an indicator for protein nitration, a posttranslational modification specific to the tyrosine amino acid which can yield protein dysfunction or turnover. A primary source and major contributor to tyrosine nitration in physiological and pathological events in vivo is through ONOO- production, a reaction by-product of NO and O2•− [65]. We described an increase in the expression of 3-NT. Similarly, other authors have found an increase in 3-NT, but in an experimental model study using pilocarpine [66]. They specifically described the fact that 3-NT accumulates in hippocampal CA3, CA1 and hilar neurons following kainate-induced status epilepticus [66].

### 4.2. Antioxidants

SOD plays a crucial role in the elimination of superoxide anion radicals (O2•−) generated from extracellular stimulants, which include ionizing radiation and oxidative insults, along with those produced in the mitochondrial matrix. In the present study, we found a significant increase in SOD activity in patients with epilepsy compared to controls. However, this was not so for glutathione, uric acid and vitamin C, that were reduced as compared with control group; these results are in agreement with previous studies, where a significantly lower activity has been found in patients with epilepsy [47].

Previous studies on SOD activity have mainly been performed in children with epilepsy on different AEDs, but they reported no significant differences in SOD activity between children with epilepsy and controls [37,67,68]. Recently, different results have shown that, after a seizure, it is possible to observe an increase of SOD activity and there is a significant decrease in SOD activity only in patients receiving lamotrigine, while valproic acid and carbamazepine were associated with weaker effects. Furthermore, after 6 months of treatment with AEDs, the activity of this antioxidant enzyme remained significantly higher in patients than in individuals in the control group [37].

The incremental SOD activity in the epileptic group subjects suggests efficient conversion of superoxide radicals to less toxic H_2_O_2_, and a reduction in the accumulation of superoxide radicals, and a compensatory mechanism reduced GPx activity or decreased the antioxidant activities of GPx and vitamin C and increased the activity of MDA, suggesting a reduced antioxidant defense capacity in epilepsy patients, and not one ameliorated by antiepileptic treatment.

### 4.3. Correlation of Oxidative Stress Parameters with Clinical Data

The results of the correlation analysis of the clinical parameters with the oxidative stress markers showed a negative relationship of MDA with the age of onset of the crises and a positive relationship with the number of drugs taken (Table 2) as the level of MDA significantly increased. The correlation with a larger number of drugs suggests that the drugs induce additional oxidative stress, while it is known that long-term use of AEDs leads to the impairment of the endogenous antioxidant system. AEDs, namely valproic acid, phenytoin, CBZ, and levetiracetam, are shown to increase lipid peroxidation and decrease the activity of the antioxidant system [69]. Similarly, a positive correlation was found with NO levels and the number of drugs. NO plays a significant role in epilepsy and epileptogenesis, since it acts as a secondary messenger, neuromodulator and neurotransmitter [70]. It has been reported that significantly elevated levels of NO are associated with the severity of epilepsy [68]. However, in the present work, the serum levels were correlated with the number of drugs used, although the NO levels were lower in epilepsy patients than in the controls (Figure 3).

The marker of oxidative stress that was most strongly associated with the severity of the epilepsy was 3-NT, which is considered as a marker of NO-dependent oxidative stress, indicating that oxidative stress is induced by seizures and AEDs, which results in seizure recurrence and drug intractability. The antioxidant system that was most negatively correlated with the severity of epilepsy was vitamin C which was associated with a greater number of seizures. Very low levels of serum vitamin C were detected; low antioxidant biomolecule levels suggest a reduced antioxidant defense capacity in epilepsy patients.

An interesting result from this study is the relationship between some oxidants and multidrug therapy. There are different studies that have related the presence of OS with anticonvulsant treatment [68]. Some of these studies were conducted on patients who were already on treatment and thus, could not conclude that the oxidative stress was a result of epilepsy or AEDs. Menon [71] compared treated and untreated patients and found that the role of AEDs in increasing oxidative stress is negligible [71]. Nevertheless, our results support that the high values of damage to lipids evaluated by MDA are closely related to the use of two or more AEDs as therapy in these patients. However, our study had a relatively small number of subjects. Studies with larger sample sizes are needed to confirm these results.

## 5. Conclusions

In summary, our results indicate altered antioxidative defenses and damage to biomolecules in patients with DRPCS. The number of AEDs influences some oxidative markers, as do the age of onset of the crisis and the frequency of seizures. The mechanism of epileptogenesis is still unclear. In spite of the advent of newer AEDs, a significant proportion of patients are refractory to treatment. Whether free radicals released after seizure episodes lead to further seizures or refractory epilepsy needs to be addressed. Considering this knowledge, in the future, it would be interesting to correlate the oxidative markers with levels of proinflammatory mediators in order to elucidate the relationship between oxidative imbalance described for these patients and these mechanisms. This study adds information to the existing literature; however, further detailed research is needed to understand the mechanism of drugs resistant epilepsy, to promote newer modalities of disease modification.

## Figures and Tables

**Table 1:** Clinical data of patients with drug resistant complex partial seizures.

No.	Age (Years)	Gender	Time Seizure Evolution (Years)	Seizure Frequency/Month	Seizure Localization	Drugs
1	21	M	17	4,5	ET	PNT, PM, CBZ
2	19	M	16	16	ET	LMG, CBZ, CLON
3	48	F	35	13	ET	CBZ, LMG
4	19	M	17	2	T	TP
5	17	M	10	7	ET	CBZ, LMG, CLB
6	22	M	16	2	T	CBZ
7	57	M	11	12	T	MV
8	38	F	18	1	ET	MV
9	46	F	46	4	ET	PBT, PNT, CBZ
10	28	F	14	90	ET	CBZ, CLON
11	30	M	18	12	T	CBZ, LMG
12	38	M	30	3	T	CBZ
13	36	M	27	2	T	CBZ, MV
14	31	F	28	120	ET	LMG, CLON
15	27	M	10	1	T	MV
16	48	M	4	30	T	LMG
17	22	F	7	30	ET	CBZ, LMG
18	16	M	9	360	T	TOP, LVT, CLON

CBZ: carbamazepine, CLB: clobazam, CLON: clonazepam, ET: extratemporal, LMG: lamotrigine, LVT: levetiracetam, PM: primidone, PBT: phenobarbital, PNT: phenytoin, MV: magnesium valproate T: temporal, TOP: topiramate.

**Table 2:** Correlation of MDA, NO, Vvtamin C and 3-NT with the time evolution of seizures, the frequency of seizures per month and the number of drugs taken by a patient.

Oxidative Stress Parameters	Age at Onset of Seizure	Frequency of Seizures per Month	Number of Drugs
MDA	−0.5500 *	0.0484	0.5351 *
AOPP	−0.1050	0.1048	−0.0291
AGEs	−0.4741	−0.3939	0.3028
NO	−0.3416	−0.3818	0.5843 *
Uric Acid	−0.1853	−0.1996	0.0529
SOD	0.1503	−0.2642	−0.2594
Glutathione	−0.1652	−0.2892	0.2182
Vitamin C	−0.2136	−0.7110 *	0.1912
3−NT	0.3878	0.7565 *	−0.0670
4−HNE	−0.0852	0.3246	0.4022

Values represent the Spearman correlation coefficients. * *p* ≤ 0.05; 3-NT: nitrotyrosine, 4-HNE: 4-hydroxy-2-nonenal, AGEs: advanced glycation end products, AOPP: advanced oxidation protein products, MDA: malondialdehyde, NO: nitric oxide, SOD: superoxide dismutase.
